# Active navigation enhances children’s spatial but not episodic memory

**DOI:** 10.3758/s13423-025-02850-y

**Published:** 2026-07-08

**Authors:** Anushari Wathiyage Don, Caren M. Walker, Lisa Musculus, Azzurra Ruggeri

**Affiliations:** 1 School of Social Sciences and Technology, Technical University of Munich, Arcisstraße 21, 80333 Munich, Germany; 2 Department of Psychology, University of California, San Diego, USA; 3 Department of Performance Psychology, German Sport University Cologne, Cologne, Germany; 4 Department of Cognitive Science, Central European University, Vienna, Austria

**Keywords:** Active control, Spatial navigation, Memory

## Abstract

This paper uses a *Yoked* experimental design to investigate the selective advantage of active spatial navigation in 4- to 10-year-old children. Participants were presented with a map game in which they were asked to find the shortest way for a monster to get across town while collecting its five monster friends on the way. Active control was associated with better recall for path length and the area between paths, whereas no differences were observed for the number of corners. Further, active navigation did not enhance episodic memory. Our results indicate that the benefits of active control during navigation are selective, supporting the encoding of global route characteristics, while leaving other detailed or incidental aspects unaffected.

## Introduction

Are you more likely to remember the grocery list if you wrote it yourself instead of having someone else do it? Will you recall different details of a landscape if you drive during a road trip rather than riding as a passenger?

Previous research has investigated the benefits of active control using memory games, in which participants study and remember objects arranged on a grid (Markant et al., 2014; Ruggeri et al., 2025; Voss, Galvan et al., 2011; Voss, Gonsalves et al., 2011). These studies often used a “yoked” design, in which participants can control the study sequence and timing in one condition, while the other involves observing the study sequence of a previous participant (Fantasia et al., 2020). Because the same content is presented, this design allows the effects of active control to be isolated. Across these studies, adults consistently showed better recognition performance for items studied under active control. This effect has been shown to persist a week after study (Voss, Galvan, et al., 2011), and to hold across a wide range of tasks and materials (Harman et al., 1999; Liu et al., 2007; Meijer & Van der Lubbe, 2011).

Recent work has investigated the emergence and developmental trajectory of the advantage of active control for memory (Partridge et al., 2015; Ruggeri et al., 2019). These findings demonstrate that the benefits of active control on object recognition appear as early as age 5 and are comparable in magnitude to those seen in adults by age 8 (Ruggeri et al., 2019). Moreover, the effects persisted a week after the initial study session (Ruggeri et al., 2019). These results have been recently replicated with samples of toddlers (Li et al., 2025), adolescents and elderly adults (Ruggeri et al., 2025), and children with autism spectrum disorder (Fantasia et al., 2020). Interestingly, these studies found minimal evidence to suggest that active control enhances spatial memory for object location.

When considering different types of spatial tasks, the impact of active engagement appears to vary (Chrastil & Warren, 2012; Meade et al., 2019; Plancher et al., 2013; Trewartha et al., 2015). Chrastil and Warren (2015) demonstrated that decision-making enhanced the acquisition of graph knowledge, evident when participants freely walked through the environment. Plancher et al. (2013) conducted a virtual driving experiment through a town and found that participants in the *Planning* and *Interaction* conditions recalled the town layout better than those in the *Passive* condition, indicating that cognitive and motor forms of active involvement enhance spatial recall. However, *Passive* participants outperformed participants in the *Interaction* group on recognition of elements encountered along the route. This suggestes that active navigation may be confined to task-relevant spatial information. Knight and Tlauka (2017) similarly showed that passive observation can support spatial learning during cognitively demanding tasks, as active control requires greater mental resources.

### The present study

The current project employs a *Yoked* design to investigate active spatial navigation in a child-friendly map-drawing game with 4- to 10-year-olds.

Understanding the effects of active learning on spatial navigation in children is critical for theoretical and practical reasons. Spatial navigation is a foundational cognitive skill that plays a crucial role in cognitive processes such as planning, decision-making, and problem-solving, all of which are essential for academic and real-world success and continue to develop during childhood. Spatial reasoning has been shown to predict future achievement in STEM fields, making it a key target for educational interventions (Uttal et al., 2013). Identifying when and how effects of active control emerge in childhood could provide valuable insights into optimizing early educational practices.

Previous research shows that active engagement in navigating a spatial environment enhances memory in young children (Feldman & Acredolo, 1979; McComas et al., 1997; Poag et al., 1983). For instance, Cohen and Cohen (1982) demonstrated that first- and sixth-graders who engaged in interrelated tasks (goal-oriented) at various stations in the classroom remembered landmark locations better than those who merely walked or performed unrelated tasks.

The present study builds on previous findings by exploring active spatial navigation with younger children and implementing a more rigorous comparison between active and passive exploration. Children were asked to find the shortest way for a monster to get to a target location while collecting its five monster friends. Children in the *Active* condition were asked to draw the most efficient path they could think of on the map, while children in the *Yoked* condition had to follow the indicated path, which corresponded to the path drawn by a previous child. We examined the effects of active control on spatial memory (e.g., path length) and episodic memory (e.g., recalling objects).

Drawing on the reviewed research, we expect that active navigation will improve accuracy in recalling the route taken, but may not affect memory for other aspects of the spatial environment (Chrastil & Warren, 2015; Plancher et al., 2013).

## Method

### Participants

Participants were 105 children aged 4 to 10 years (46 girls; *M* = 92.52 months; *SD *= 18.98 months). Fourteen additional children were excluded due to major difficulties in understanding the memory questions or the task (*n* = 5), distraction (*n* = 3), experimental error (*n* = 2), diagnosed with neurodevelopmental conditions (*n* = 2), or being outside the intended age range (*n* = 2). All other data were retained, as no predefined exclusion criteria for memory were defined. To estimate the sample size prior to data collection, we performed a power analysis on a simulated dataset, which indicated that at least 80 children across conditions were required to detect a difference between conditions with 80% power and 0.8 estimated effect size, with a 0.05 criterion for statistical significance. We tested more children than suggested by the power analysis to ensure representation across age within the sample. Children were recruited and tested in daycare centers and public parks in Italy.

Ethics approval was obtained from the ethics committee of the Max Planck Institute for Human Development. Prior to participation, written informed consent was obtained from the children’s parents or legal guardians. The children received a small present to thank them for their participation.

### Design

**Fig. 1:**
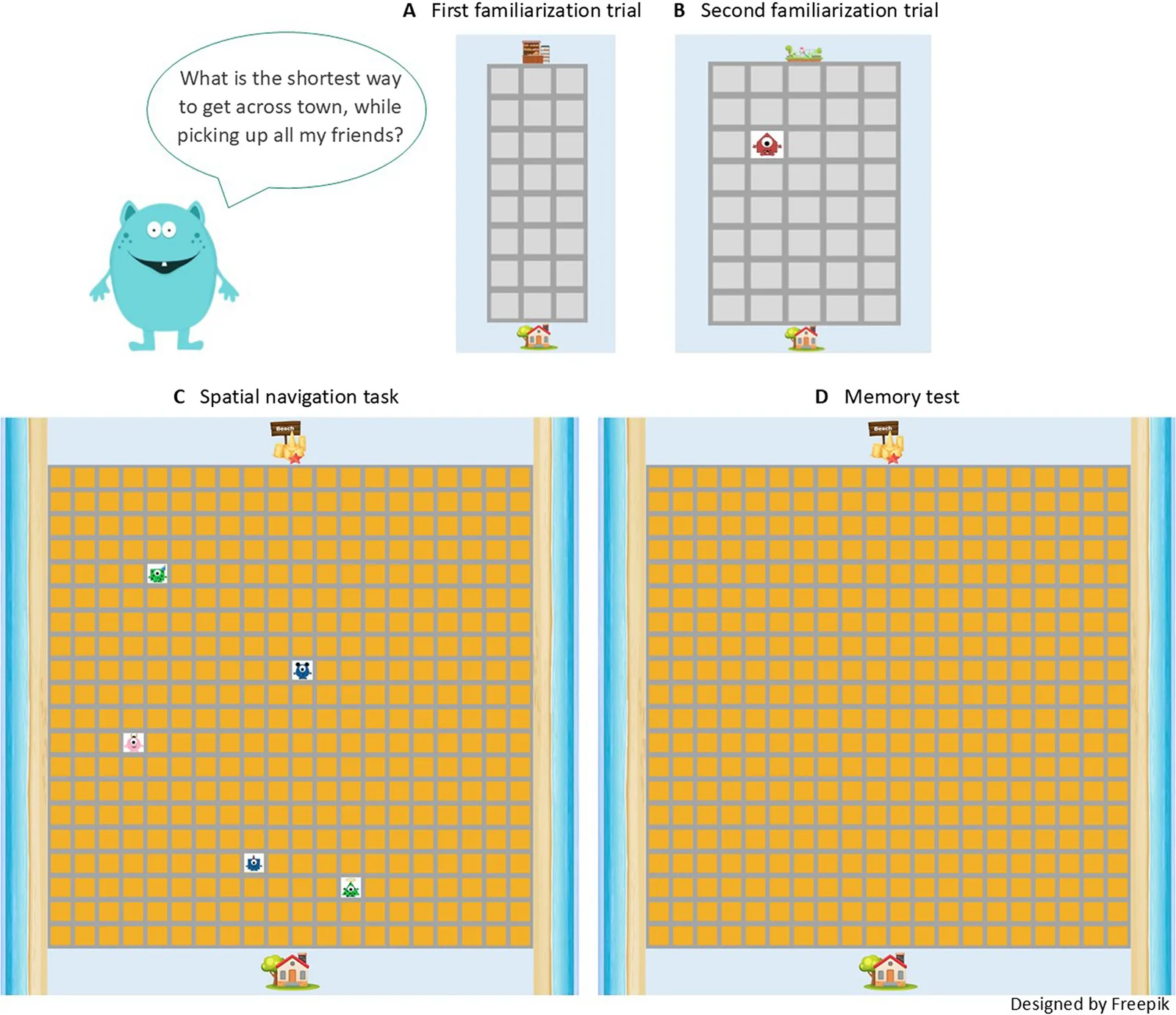
Overview of the simplified procedure. **A–B** Maps used for the familiarization trials. **C** Illustration of one of the maps (beach scenario) participants were presented with during the spatial navigation task. The experimenter placed the five monster friends one by one on their position on the grid before children were asked to draw the shortest way for Gigi to get from home to the beach/school (*Active* condition) or to follow the path with a toy car (*Yoked* condition), making sure they picked up Gigi’s five monster friends. **D** Depiction of the map participants were presented in the memory test

Children were tested in a spatial navigation game in which they were tasked with finding the shortest way for a monster named *Gigi* to get across town (see Fig. 1). On the way, *Gigi* should collect its five monster friends, who carried one object each with them. The experimental session consisted of three phases: familiarization, spatial navigation task, and memory test. Each session took approximately 10 to 15 minutes.

### Procedure

#### Familiarization

Children were first presented with two maps to introduce them to the aim of the task and the procedure. In the first familiarization trial, the map consisted of a 3 × 8 grid, with Gigi’s home on one side and a bakery on the other (see Fig. 1A). Children were instructed to help Gigi find the shortest way from Gigi’s home to the bakery by drawing the path with a marker directly along the gray lines between the cells of the map. They were then told that every time they crossed a street, they would lose stars (out of an initial endowment of 10 stars) and that the more stars they had left at the end of the game, the bigger the prize they would win. While drawing the path, a counter on the table reminded them of the number of stars left. Importantly, the counter was used primarily as a visual cue to choose the shortest path and help children to focus on route efficiency in a child-friendly way. Note that 61% of the children drew a path that followed the gray lines in a direct route from Gigi’s home to the bakery, without taking unnecessary turns. Gigi’s home and the bakery are positioned between two corner points of the grid on the map. For the evaluation, we used the corners of the cells to define the starting and end points of the route, and assessed whether participants drew a direct path along the gray lines between these two points.

After that, children were presented with the second familiarization trial. This map consisted of a 5 × 8 grid, with Gigi’s home on one side and a playground on the other (see Fig. 1B). This time, the experimenter placed a colored card on the grid, illustrating a second, different monster. Children were told their goal was to help Gigi find the shortest way from home to the playground, but this time, they drew a path that passed by Gigi’s friend. They were reminded of the rules again. Note that 66% of the children successfully drew an efficient route along the gray lines without taking unnecessary turns while collecting the monster friend on the way from Gigi’s home to the playground. At the end of the second familiarization, the experimenter highlighted that participants should always try to find the shortest way and to draw their route only along the gray lines between the cells.

#### Spatial navigation task

**Fig. 2:**
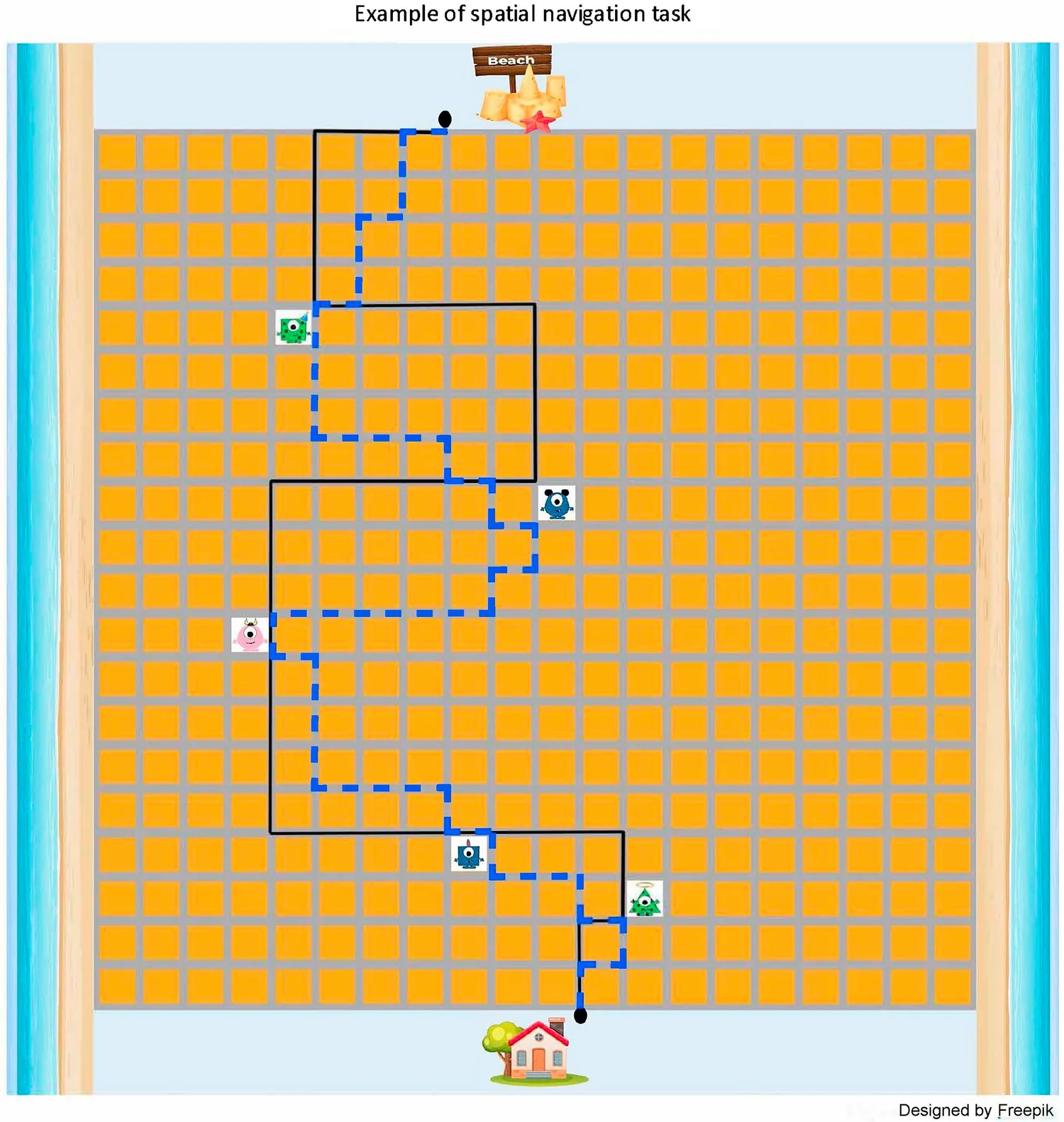
Example routes for the spatial navigation task. Depiction of a possible efficient route (black, solid) and a route drawn by a participant in the *Active *condition (blue, dashed)

After the familiarization, children were presented with a new map consisting of a 20 × 20 grid, on which the experimenter placed five colored cards illustrating Gigi’s monster friends (always in the same position on the grid; see Fig. 1C). We manipulated whether children were assigned to the *Active* or the *Yoked* condition in a between-subject design. In the *Active* condition, children were asked to draw the shortest way for Gigi to get from home to the beach/school (scenarios were counterbalanced across participants), making sure they picked up Gigi’s five monster friends (see Fig. 2). A monster friend was considered to be collected when the path reached the corner of the grid cell in which the monsters were located. When children passed by one of the five monster friends, the experimenter picked up the card, told them the name of that monster, and showed them the special object the monster friend was carrying twice (e.g., “See, this is Adam. He is carrying a cake with him.”). After that, the experimenter put the card aside so the children could not see it. The experimenter also ensured that children picked up all five monster friends and verbally reminded children who forgot to collect a friend to pick up all friends. In the *Yoked* condition, children were shown the way from Gigi’s home to the beach/school that a previous participant had generated in the *Active* condition, and were asked to follow the same path with a toy car.

#### Memory test

The memory test consisted of two main tasks. In the spatial memory task, children were presented with an empty beach/school map only showing the starting and end point, identical to the one they had been presented with for the spatial navigation task (see Fig. 1D). They were asked to draw the way they had previously taken from Gigi’s home to the beach/school, as they remembered it. In the episodic memory task, children were asked to recognize and point to Gigi’s monster friends among a set of 15 monsters (including the five monster friends mixed with 10 distractors), and to recall the objects the monster friends were carrying.

## Results

The anonymized database containing the raw data and regression results are published in the Open Science Framework repository.

### Spatial navigation task (*Active* condition)

Approximately 85% of the participants in the *Active* condition drew a direct path from Gigi’s home to the beach/school without ever backtracking or retracing their route. Participants drew paths with an average length of 46.47 segments (*SD* = 4.77 segments), including on average 13.98 corners (*SD* = 4.37 corners).

For path length, a linear regression indicated an Age (in months) effect, *F*(1, 51) = 4.29, *p* = *.*043, *R*^2^ = *.*078. However, this effect was not robust when using heteroskedasticity-robust standard errors (*p* = *.*191). For descriptive purposes, we also report group means based on a median split of the age range. Older children (96 to 125 months; *n* = 27) drew slightly shorter paths (*M* = 46.04, *SD* = 3.30) compared with younger children (58 to 95 months; *n* = 26), who generated paths of an average length of 46.92 segments (*SD* = 5.97).

We found a main effect of Age on the number of corners, *F*(1, 51) = 12.46, *p* < *.*001, *R*^2^ = *.*196. Older children (96 to 125 months) drew paths including, on average, 12.41 corners (*SD* = 3.08 corners), whereas younger children (58 to 95 months) drew paths including, on average, 15.62 corners (*SD* = 4.94 corners).

### Spatial memory task

To evaluate the accuracy of children’s memory of the path they had previously drawn (*Active* condition) versus followed (*Yoked* condition), we considered three distinct measures:

1. The absolute difference in path length, measured as the number of grid segments by which the recalled path differed;
2. The absolute difference in number of corners, measured as the difference in the number of directional changes between the original and remembered paths; and
3. The area between paths, calculated as the number of grid cells (i.e., square units on the map) between the original and remembered paths.

In three cases for the area measure, the raters could not reach agreement; they resolved this by averaging their codings. Lower values on the three measures indicate a greater spatial memory accuracy. Each of these measures provides complementary information about how participants represented and reproduced the path. Path length reflects deviations in overall path distance, which may suggest that participants prioritized route efficiency and actively monitored the distance during navigation. Corners capture participants’ memory for the structure of the path, which represents the memory for directional changes. Finally, the area measure represents differences in the spatial layout between the encoded and reproduced paths, and reflects an ability to reconstruct the overall layout.

**Fig. 3:**
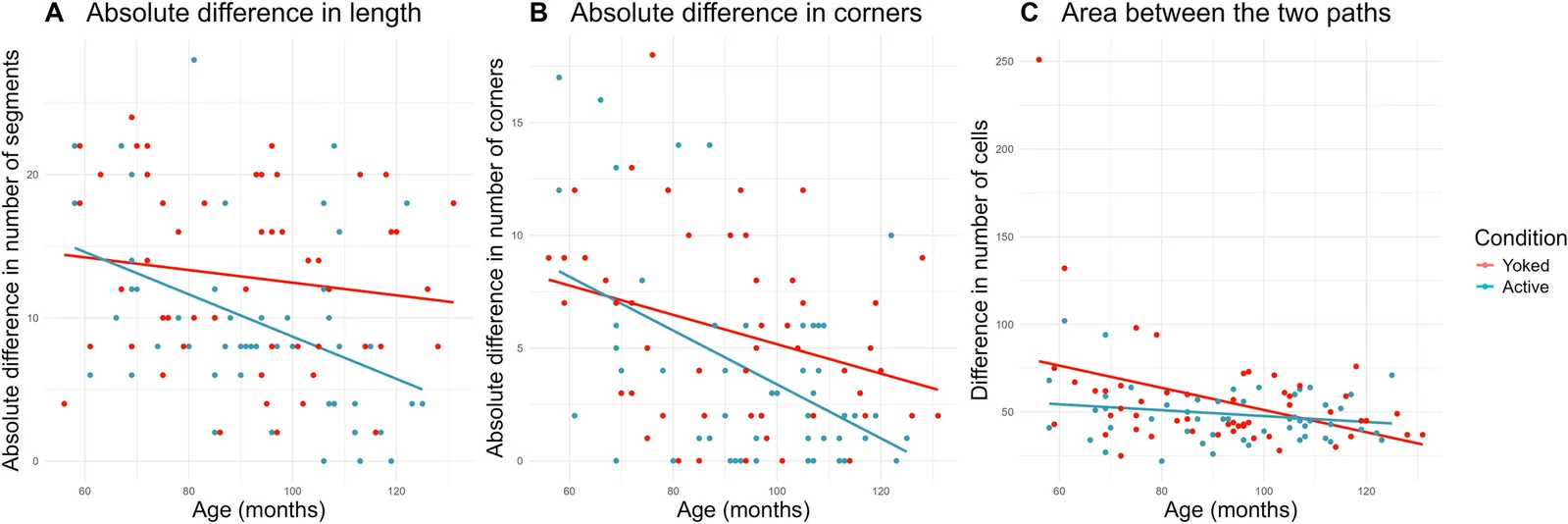
Overview of memory results for the spatial measures. Individual points represent participants; solid lines show separate linear regressions fitted for each condition. Lower values indicate greater memory accuracy. **A** Absolute difference in path length. **B** Absolute difference in corners. **C** Area between the paths in the *Active* (blue) and *Yoked* (red) conditions as a function of age

We performed a series of analyses of variance (ANOVAs), with each of the measures as dependent variables, Condition (*Active* vs. *Yoked*) as the main predictor, and including Age (in months) and their interaction as further predictors. Below, we report the best-fitting models for each of the measures considered (see Fig. 3).

#### Absolute difference in length

On average, the path children remembered drawing in the *Active* condition differed in length by 9.58 segments (*SD* = 6.45 segments) compared with the one they had actually drawn. In contrast, in the *Yoked* condition, children’s remembered path differed by about 12.85 segments (S*D* = 6.22 segments) from the one they had actually followed. The analyses revealed a significant main effect of Age, *F*(1, 102) = 8.49, *p* = *.*004, partial *η*^2^ = *.*08, and Condition, *F*(1, 102) = 6.28, *p* = *.*014, partial *η*^2^ = *.*06, on the difference in length. Older children were estimated to remember paths closer in length to the original path, with an estimated decrease of 0.092 segments in error per month (*B* = −0.092, *SE* = 0.032, *p* = *.*004), equivalent to approximately 1.1 segments per year. Including the interaction term did not significantly improve model fit, *χ*^2^(1) = 2*.*76*, p* = *.*097.

#### Absolute difference in corners

On average, the path children remembered drawing in the *Active* condition differed by 4.11 corners (*SD* = 4.46 corners) relative to the one they had actually drawn. In the *Yoked* condition, children’s remembered path differed by about 5.75 corners (*SD* = 4.07 corners) from the one they had actually followed. The analyses revealed a significant main effect of Age, *F*(1, 102) = 19.73, *p < .*001, partial *η*^2^ = *.*16, indicating that older children remembered the number of corners more accurately than younger children. For every additional month of age, children’s error in corner recall decreased by 0.090 corners (*B* = −0.090, *SE* = 0.020, *p < .*001), equivalent to approximately 1.1 corners per year. There was no significant main effect of Condition, *F*(1, 102) = 3.22, *p* = *.*075, partial *η*^2^ = *.*03. Including the interaction term did not significantly improve model fit, χ^2^(1) = 1*.*84*, p* = *.*175.

#### Area between the two paths

In the *Active* condition, the area between both paths comprised, on average, 48.68 grid cells (*SD* = 15.40), compared with 56.75 grid cells (*SD* = 33.48) in the *Yoked* condition. The analyses revealed a significant main effect of Age, *F*(1, 101) = 13.18, *p < .*001, partial *η*^2^ = *.*12, and Condition, *F*(1, 101) = 4.29, *p* = *.*041, partial *η*^2^ = *.*04. The interaction was not significant, *F*(1, 101) = 3.32, *p* = *.*071, partial *η*^2^ = *.*03. Each additional month of age was associated with a reduction of 0.633 grid cells in area error (*B* = −0.633, *SE* = 0.174, *p* < .001), equivalent to approximately 7.6 grid cells per year.

### Episodic memory task

To measure performance on the episodic memory task, we considered two distinct measures:

1. The number of objects participants correctly recalled, and
2. The number of monster friends correctly recognized among the distractors.

For each measure, we fit binomial generalized linear models with the number of correct responses (out of five) as the dependent variable, Age (in months), Condition (*Active* vs. *Yoked*), and their interaction as predictors. Model comparisons showed that the interaction did not improve model fit for object recall, *χ*^2^(1) = 1.04, *p* = *.*307; or monster recognition, *χ*^2^(1) = 0.82, *p* = *.*364, and we therefore report the additive models. On average, participants recalled 3.36 objects (*SD =* 1.16) in the *Active* condition, compared with 3.31 objects (*SD* = 0.88) in the *Yoked* condition. The effect of Condition was not significant, *χ*^2^(1) = 0.01, *p* = *.*907, with the odds of object recall differing only minimally between groups (*OR* = 1.02). The odds of correctly recalling objects were estimated to increase slightly with age (*OR* = 1.104 per year), but this effect did not reach significance, *χ*^2^(1) = 2.83, *p* = *.*093.

On average, participants recognized 3.62 monsters (*SD* = 1.06) in the *Active *condition, and 3.57 monsters (*SD* = 0.94) in the *Yoked* condition. Neither Age, *χ*^2^(1) = .003, *p* = *.*956, nor Condition, *χ*^2^(1) = 0.08, *p* = *.*782, were significant predictors of monster recognition performance. Odds of correct recognition were estimated to be slightly higher in the *Active* condition (*OR* = 1.06), and to decrease slightly with age (*OR* = 0.997 per year), but neither effect was significant.

## General discussion

The present study investigated whether active control impacts spatial and episodic memory in children using a *Yoked* experimental paradigm.

We examined three spatial measures to assess the memory for different elements of the route in both conditions. Importantly, the spatial navigation task in the *Active* condition additionally provides insight into the strategies children used to approach the navigation task, such as route efficiency and the degree to which children relied on segmented forms of spatial navigation.

Our findings revealed that children in the *Active* condition exhibited better spatial memory for path length and the area between paths. Memory for the number of corners did not differ between conditions. These findings partially replicate previous work with adults demonstrating that active navigation enhances spatial memory (Chrastil & Warren, 2015; Meade et al., 2019; Plancher et al., 2013).

One potential reason for the observed advantages is that active control involves elements of decision-making processes, which may support encoding spatial information by enhancing participants’ attention or engaging in more complex cognitive processes (see Chrastil & Warren, 2015; Markant et al., 2016; Plancher et al., 2013). Active control allows participants to regulate and adapt the pace and sequence of navigation to align with their attentional state. In contrast, participants in the *Yoked* condition may be more likely to experience distraction, such as mind-wandering or processing previously encountered information, which leads to less effective encoding of new spatial information (Markant et al., 2016). Another reason could be that actively generating a route can strengthen spatial representation. Engaging in actions like drawing the path can create additional contextual elements connected to the learned information, further enhancing later recall (see Markant et al., 2016, for a review; Hommel, 2004).

Our findings are consistent with previous studies, but also suggest that active control may selectively enhance memory, depending on task demands, as it did not enhance memory for the number of corners. One possible explanation could be that turns reflect local details, which are more difficult to remember than broader route characteristics. Our analyses showed that corner accuracy improved with age, suggesting that memory for turns may depend more on developmental changes. These age-related differences may reflect the ongoing development of cognitive abilities related to executive functioning and self-regulation (Roebers, 2017).

In the *Active* condition, we also observed age-related differences in how children generated paths. Younger children tended to generate paths with more corners, despite showing similar overall path length compared with older children. This may suggest that they used a more segmented navigation strategy, involving more directional changes. In real-world settings, each additional change of direction can increase navigational error. Thus, routes with more corners may also be harder to remember; therefore, in our drawing-based task, additional corners may similarly increase the opportunities for errors. Visual inspection suggested a developmental shift toward fewer directional changes that may emerge around 85–90 months. This aligns with research that has discussed significant improvements in cognitive resources during what is often referred to as the “five-to-seven-year shift” (Ruggeri et al., 2019). During this period, children experience notable advancements in their ability to control attention and regulate behavior, which may help explain the trend toward simpler paths in older children.

The effects of active control did not extend to episodic memory performance. Prior research suggests that active engagement selectively enhances memory for task-relevant spatial information but not for elements encountered along the route (Brooks et al., 1999; Plancher et al., 2013). In our study, children were primarily engaged with the spatial elements of the task (e.g., drawing paths) and did not necessarily engage with the episodic information. Further, the cognitive demands of spatial navigation may have limited the encoding of these incidental details. Children performed well in both tasks and conditions, with no age difference observed. However, there was no indication of substantial ceiling effects. Compared with studies that include more complex and immersive episodic memory tasks (see Plancher et al., 2013; Ruggeri et al., 2019), our task may have been less engaging, and therefore future research could vary the complexity and engagement level of the spatial navigation tasks and examine a broader range of episodic memory tasks to determine under what conditions active navigation enhances or hinders episodic memory.

Overall, our findings suggest that the benefits of active control may be selective, supporting the encoding of broader route characteristics (efficiency and layout) more than detailed features such as individual turns. Importantly, our study extends previous findings obtained with complex virtual navigation tasks by demonstrating that the effects of active control can also be observed with simple procedures. This simplicity reduces testing time and cognitive load, making it more suitable for younger populations, and increases the feasibility of running studies in educational and field settings where technical resources or time are limited. The findings of our study may have implications for early education, where opportunities to actively explore routes may strengthen broader spatial reasoning skills, which are essential for navigation and learning in everyday contexts. For example, in outdoor play settings, children can be allowed to actively plan their own route during treasure hunts that are connected to learning activities. Similarly, maze- or map-based activities could encourage children to actively test different strategies to reach a target. These activities can be implemented in various formats, including paper-and-pencil, tablet applications, or building blocks, providing opportunities for active spatial exploration from an early age onward.

## Conclusion

To conclude, this study demonstrates that active control during navigation leads to a selective advantage for spatial memory performance in children.

Our findings contribute to the growing body of work aimed at understanding the development of memory. The evidence that active control selectively enhances spatial memory suggests that even at an early age, children can benefit from environments that encourage self-directed exploration. Spatial memory plays a central role in daily tasks, from navigating familiar environments to organizing thoughts and ideas in structured ways. Strengthening these abilities early on might support broader cognitive development.

## Data Availability

Samples of the study materials, the raw data, and regression results are archived in the Open Science Framework repository.

## Code availability

Code for data analysis will be shared upon request.

## References

[CR1] Brooks, B. M. (1999). The specificity of memory enhancement during interaction with a virtual environment. *Memory,**7*(1), 65–78. 10.1080/741943713 10645373

[CR2] Chrastil, E. R., & Warren, W. H. (2012). Active and passive contributions to spatial learning. *Psychonomic Bulletin & Review,**19*, 1–23. 10.3758/s13423-011-0182-x 22083627

[CR3] Chrastil, E. R., & Warren, W. H. (2015). Active and passive spatial learning in human navigation: Acquisition of graph knowledge. *Journal of Experimental Psychology: Learning, Memory, and Cognition,**41*(4), 1162–1178. 10.1037/xlm0000082 25419818

[CR4] Cohen, S., & Cohen, R. (1982). Distance estimates of children as a function of type of activity in the environment. *Child Development,**53*(3), 834–837. 10.2307/1129400

[CR5] Fantasia, V., Markant, D. B., Valeri, G., Perri, N., & Ruggeri, A. (2020). Memory enhancements from active control of learning in children with autism spectrum disorder. *Autism,**24*(8), 1995–2007. 10.1177/1362361320931244 32579025

[CR6] Feldman, A., & Acredolo, L. (1979). The effect of active versus passive exploration on memory for spatial location in children. *Child Development,**50*(3), 698–704. 10.2307/1128935 498846

[CR7] Harman, K. L., Humphrey, G. K., & Goodale, M. A. (1999). Active manual control of object views facilitates visual recognition. *Current Biology,**9*(22), 1315–1318. 10.1016/S0960-9822(00)80053-6 10574764

[CR8] Hommel, B. (2004). Event files: Feature binding in and across perception and action. *Trends in Cognitive Sciences,**8*(11), 494–500. 10.1016/j.tics.2004.08.007 15491903

[CR9] Knight, M. J., & Tlauka, M. (2017). Interactivity in map learning: The effect of cognitive load. *Spatial Cognition & Computation,**17*(3), 185–198. 10.1080/13875868.2016.1211661

[CR10] Li, Y.-L., Poli, F., & Ruggeri, A. (2025). Active control over exploration improves memory in toddlers. *Proceedings of the Royal Society B: Biological Sciences,**292*(2039), Article 20242555. 10.1098/rspb.2024.2555 PMC11777551 39876734

[CR11] Liu, C. H., Ward, J., & Markall, H. (2007). The role of active exploration of 3D face stimuli on recognition memory of facial information. *Journal of Experimental Psychology: Human Perception and Performance,**33*(4), 895–904. 10.1037/0096-1523.33.4.895 17683235

[CR12] Markant, D., DuBrow, S., Davachi, L., & Gureckis, T. M. (2014). Deconstructing the effect of self-directed study on episodic memory. *Memory & Cognition,**42*, 1211–1224. 10.3758/s13421-014-0435-9 24941938 PMC4281964

[CR13] Markant, D. B., Ruggeri, A., Gureckis, T. M., & Xu, F. (2016). Enhanced memory as a common effect of active learning. *Mind, Brain, and Education,**10*(3), 142–152. 10.1111/mbe.12117

[CR14] McComas, J., Dulberg, C., & Latter, J. (1997). Children’s memory for locations visited: Importance of movement and choice. *Journal of Motor Behavior,**29*(3), 223–229. 10.1080/00222899709600837 12453781

[CR15] Meade, M. E., Meade, J. G., Sauzeon, H., & Fernandes, M. A. (2019). Active navigation in virtual environments benefits spatial memory in older adults. *Brain Sciences,**9*(3), 47. 10.3390/brainsci9030047 30813536 PMC6468686

[CR16] Meijer, F., & Van der Lubbe, R. H. (2011). Active exploration improves perceptual sensitivity for virtual 3d objects in visual memory. *Vision Research,**51*(23/24), 2431–2439. 10.1016/j.visres.2011.09.013 22005389

[CR17] Partridge, E., McGovern, M. G., Yung, A., & Kidd, C. (2015). Young children’s self-directed information gathering on touchscreens. In D. C. Noelle, R. Dale, A. S. Warlaumont, J. Yoshimi, T. Matlock, C. D. Jennings, & P. P. Maglio (Eds.), *Proceedings of the 37th Annual Conference of the Cognitive Science Society* (pp. 1835–1840). Cognitive Science Society.

[CR18] Plancher, G., Barra, J., Orriols, E., & Piolino, P. (2013). The influence of action on episodic memory: A virtual reality study. *Quarterly Journal of Experimental Psychology,**66*(5), 895–909. 10.1080/17470218.2012.722657 23025821

[CR19] Poag, C. K., Cohen, R., & Weatherford, D. L. (1983). Spatial representations of young children: The role of self- versus adult-directed movement and viewing. *Journal of Experimental Child Psychology,**35*(1), 172–179. 10.1016/0022-0965(83)90077-2 6827216

[CR20] Roebers, C. M. (2017). Executive function and metacognition: Towards a unifying framework of cognitive self-regulation. *Developmental Review,**45*, 31–51. 10.1016/j.dr.2017.04.001

[CR21] Ruggeri, A., Markant, D. B., Gureckis, T. M., Bretzke, M., & Xu, F. (2019). Memory enhancements from active control of learning emerge across development. *Cognition,**186*, 82–94. 10.1016/j.cognition.2019.01.010 30769196

[CR22] Ruggeri, A., Li, Y.-L., Battisti, A., & Markant, D. B. (2025). Active control of learning enhances memory across the lifespan. *Journal of Educational Psychology,**117*(3), 529–540. 10.1037/edu0000927

[CR23] Trewartha, K. M., Case, S., & Flanagan, J. R. (2015). Integrating actions into object location memory: A benefit for active versus passive reaching movements. *Behavioural Brain Research,**279*, 234–239. 10.1016/j.bbr.2014.11.043 25476567

[CR24] Uttal, D. H., Meadow, N. G., Tipton, E., Hand, L. L., Alden, A. R., Warren, C., & Newcombe, N. S. (2013). The malleability of spatial skills: A meta-analysis of training studies. *Psychological Bulletin,**139*(2), 352–402. 10.1037/a0028446 22663761

[CR25] Voss, J. L., Galvan, A., & Gonsalves, B. D. (2011). Cortical regions recruited for complex active-learning strategies and action planning exhibit rapid reactivation during memory retrieval. *Neuropsychologia,**49*(14), 3956–3966. 10.1016/j.neuropsychologia.2011.10.012 22023912 PMC3223278

[CR26] Voss, J. L., Gonsalves, B. D., Federmeier, K. D., Tranel, D., & Cohen, N. J. (2011). Hippocampal brain-network coordination during volitional exploratory behavior enhances learning. *Nature Neuroscience,**14*(1), 115–120. 10.1038/nn.2693 21102449 PMC3057495

